# The Mediating Role of Job Rotation Between Clinical Leadership Skills and Clinical Decision‐Making Among Jordanian Nurses

**DOI:** 10.1155/jonm/3436144

**Published:** 2026-06-27

**Authors:** Luma Ghazi Alzamel, Samar Thabet Jallad, Khitam Alsaqer, Maram Taher Alghabbashi

**Affiliations:** ^1^ Nursing Leadership and Management, Department of Nursing, Nursing Faculty, Applied Science Private University, Amman, Jordan, asu.edu.jo; ^2^ Nursing Education, Department of Nursing, Faculty of Health Professions, Al-Quds University, Jerusalem, State of Palestine, alquds.edu; ^3^ Community and Mental Health Nursing Department, Faculty of Nursing, Zarqa University, Zarqa, Jordan, jadara.edu.jo; ^4^ Faculty of Nursing, Umm Al-Qura University, Makkah, Saudi Arabia, uqu.edu.sa

**Keywords:** clinical decision-making, clinical leadership, job rotation, Jordan, mediation analysis, nurses

## Abstract

**Background:**

Nurses in resource‐limited settings like Jordan require strong leadership and clinical judgment. Job rotation is believed to improve adaptability and self‐efficacy, but its effect on clinical decision‐making is unclear.

**Purpose:**

This study aimed to examine whether job rotation mediates the relationship between clinical leadership skills and clinical decision‐making among nurses.

**Methods:**

A cross‐sectional correlational study was conducted among 395 registered nurses employed in Jordanian governmental hospitals. Participants were recruited using a proportional random sampling technique. Data were collected using validated questionnaires assessing clinical leadership skills, job rotation, and clinical decision‐making. Statistical analyses, including ANOVA and regression analysis, were performed to examine relationships among the study variables. Structural equation modeling (SEM) was additionally used to assess mediation effects.

**Findings:**

The results showed significant differences between clinical decision‐making and gender, marital status, and educational level. Clinical leadership significantly predicted decision‐making (*β* = 0.433, 95% CI [0.331, 0.535], *t* = 8.226, *p* < 0.001). In contrast, job rotation indicated no significant direct effect (*β* = 0.000, 95% CI [−0.052, 0.051], *t* = −0.010, *p* = 0.992) and did not mediate the relationship between leadership and decision‐making (*β* = 0.008, 95% CI [−0.041, 0.057], *t* = 0.325, *p* = 0.745).

**Conclusion:**

Leadership skills were significantly associated with nurses’ clinical decision‐making, and leadership development may play a more substantial role in clinical decision‐making than job rotation within this sample.

**Implication for Nursing Management:**

Job rotation implementation has to be intentional, by putting real effort into structured training, mentoring, and embedding workplace learning into daily routines, which improves decision‐making quality, increases professional confidence, and ultimately enhances patient outcomes.

## 1. Introduction

Nurses have a critical role in maintaining healthcare quality as primary caregivers and decision‐makers in dynamic, complex clinical environments [1]. The continuous evolution of healthcare systems, characterized by rapid scientific progress, complex care demands, and high patient turnover [2], has intensified nurses’ workloads, often resulting in stress, burnout, and reduced satisfaction [3]. Clinical leadership skills (CLS) are essential for promoting effective clinical decision‐making (CDM), improving patient safety, and enhancing healthcare quality outcomes among nurses. Nurse leaders with strong leadership competencies are more capable of making timely, evidence‐based clinical decisions in complex healthcare environments. Recent evidence has highlighted the critical role of nursing leadership in maintaining patient safety and strengthening clinical performance within healthcare systems [4]. Additionally, individual professional characteristics, such as self‐concept and confidence, have been shown to significantly influence nurses’ CDM abilities [5]. Therefore, strengthening leadership competencies among nurses remains a strategic priority for healthcare organizations. Conversely, poor leadership development contributes to disengagement, limited career progression, and workplace fatigue [6]. Consequently, developing resilient and efficient workforce strategies has become a crucial priority for sustaining healthcare outcomes.

One promising approach in healthcare is job rotation (JR), which has emerged as an important nursing workforce strategy aimed at enhancing professional competency and improving adaptability within complex healthcare environments [7]. In nursing practice, JR allows nurses to work across different clinical departments and patient care settings, thereby increasing clinical exposure and broadening practical experience [8]. Such rotational experiences may strengthen clinical judgment, interdisciplinary collaboration, communication skills, and the ability to respond effectively to diverse and complex patient care situations (Heinen et al., 2019). Previous nursing studies have further suggested that exposure to varied clinical environments contributes to professional development, workforce engagement, resilience, and leadership competency development among nurses [9]. These competencies are considered essential for strengthening effective clinical leadership and supporting evidence‐based CDM in healthcare settings (Chatterjee et al., 2023).

Within Jordan’s healthcare system, nurses face unique pressures, including workforce shortages, resource constraints, and a growing burden of chronic diseases. In such contexts, structured JR may enhance professional development, promote adaptability, and support organizational objectives like retention and leadership cultivation [10–12]. These ongoing pressures have intensified the demand for effective workforce development strategies that enhance professional competency, adaptability, and staff retention within healthcare organizations. In this context, structured JR programs may serve as an important organizational strategy to promote professional growth, strengthen clinical competencies, improve workforce flexibility, and support leadership development among nurses [10–12]. By exposing nurses to diverse clinical settings and patient care experiences, JR may enhance their ability to respond effectively to complex and rapidly changing healthcare environments.

This perspective is consistent with experiential learning theory (ELT), which proposes that professional knowledge and competencies are developed through continuous cycles of concrete experience, reflective observation, conceptualization, and active experimentation [13]. Within nursing practice, JR may function as an experiential learning mechanism that enables nurses to broaden their clinical exposure, engage in diverse patient care situations, and develop practical leadership competencies through active participation across different healthcare settings.

From a theoretical perspective, ELT suggests that experiential exposure facilitates the transformation of theoretical leadership knowledge into practical clinical competence. Consequently, JR may theoretically mediate the relationship between CLS and CDM by providing nurses with opportunities to apply leadership behaviors in real‐world clinical contexts, reflect on their experiences, and develop stronger decision‐making competencies through practice‐based learning [13].

**FIGURE 1:**
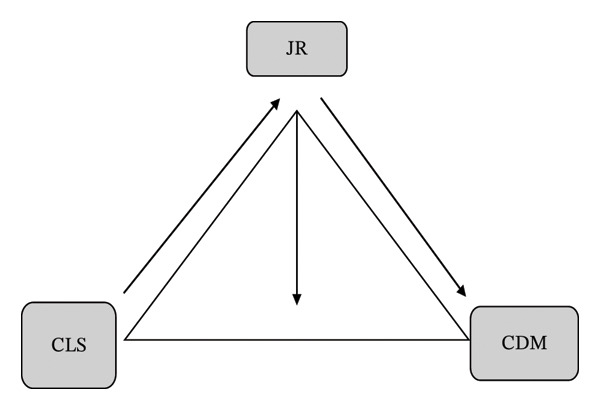
The researchers’ proposed conceptual framework of the study. Footnote: JR: job rotation, CDM: clinical decision‐making, CLS: clinical leadership skills.

Despite increasing attention to nursing leadership and CDM, limited research has examined the mediating role of JR within this relationship, particularly in Middle Eastern healthcare contexts. In Jordan and similar Middle Eastern healthcare systems, limited evidence exists regarding the mediating role of JR between CLS and CDM among nurses. Most existing evidence concerning JR originates from industrial and corporate sectors rather than nursing and healthcare environments. Addressing this research gap may provide important insights into workforce development strategies that foster leadership competencies, strengthen CDM abilities, improve job satisfaction, and support professional growth among Jordanian nurses [9]. Strengthening these dimensions may ultimately contribute to workforce sustainability, healthcare quality improvement, and better patient outcomes. Therefore, this study is based on a conceptual model in which CLS represent the independent variable, CDM represents the dependent variable, and JR functions as a mediating variable (CLS ⟶ JR ⟶ CDM) (Figure 1).

### 1.1. Aim of the Study

This study aimed to examine the mediating role of JR in the relationship between CLS and CDM among nurses working in Jordanian governmental hospitals.

### 1.2. The Study Questions

1. What are the demographic and occupational characteristics of participating nurses?
2. What are the associations between CLS, JR, and CDM among participating nurses?
3. Does JR mediate the relationship between CLS and CDM among participating nurses?

## 2. Method

### 2.1. Study Design

This study utilized a quantitative, cross‐sectional, correlational approach adhering to STROBE criteria to investigate whether JR mediates the relationship between leadership skills and CDM among nurses. Data were collected at a single point, allowing us to assess how these variables interact within current hospital practice.

### 2.2. Setting and Sample

The study was conducted in three major governmental hospitals in Jordan: Al Bashir Hospital, Jordan University Hospital, and Al Zarqa Governmental Hospital. These hospitals provide secondary and tertiary healthcare services and include a variety of clinical departments, such as emergency departments, intensive care units (ICUs), pediatric units, and general medical–surgical wards. The total target population consisted of 2648 registered nurses employed across the participating hospitals, including 1500 nurses from Al Bashir Hospital, 800 nurses from Jordan University Hospital, and 348 nurses from Al Zarqa Governmental Hospital.

The required sample size was calculated using the online eSoft sample size calculator based on a 95% confidence level, 5% margin of error, and a statistical power level of 0.80, assuming a medium effect size appropriate for correlational and regression analyses. The calculation indicated that a minimum sample size of 336 participants was required to achieve adequate statistical precision and representativeness.

A proportional random sampling technique was employed to ensure proportional representation from each participating hospital according to the number of registered nurses employed at each institution. The number of participants selected from each hospital was determined proportionally based on its nursing workforce size. Eligible nurses working in the selected clinical departments were identified through hospital nursing administration records. Subsequently, nurses were randomly approached and invited to participate in the study.

Data were collected electronically using Google Forms. The survey link was distributed to eligible participants through nursing supervisors and official institutional communication channels within the participating hospitals. A total of 395 registered nurses completed the questionnaire, exceeding the minimum required sample size and thereby enhancing the statistical adequacy of the study. However, the use of an online self‐administered questionnaire may have introduced selection bias, as participation may have been limited to nurses with greater internet accessibility and technological familiarity.

### 2.3. Inclusion and Exclusion Criteria

Eligibility criteria included being a licensed registered nurse employed on a full‐time basis with a minimum of 6 months of clinical experience. Nursing students and healthcare professionals from non‐nursing disciplines were excluded from participation.

### 2.4. Data Collection Instruments and Procedures

Data collection was conducted via a structured, self‐administered survey distributed through Google Forms. The instrument was organized into four distinct sections.

The first section gathered demographic details such as gender, age, marital status, educational background, departmental affiliation, years of clinical experience, and frequency of JR (categorized as once, twice, or three times or more).

The second section utilized the clinical leadership survey (CLS) developed by Chappell et al., a validated tool comprising 15 items that assess leadership competencies, including outcome influence, teamwork, and communication skills. Responses were captured on a 5‐point Likert scale (ranging from never to always), with higher scores indicating stronger leadership potential [14].

The third section, the survey implemented the Clinical Decision‐Making in Nursing Scale (CDMNS), also a 15‐item instrument, which aligns with key components of the clinical reasoning cycle (assessment, diagnosis, planning, and intervention). Each item was rated on a 5‐point Likert scale. The validity of this instrument has been established in various healthcare settings [15].

The fourth section featured the Job Rotation Survey by Ho et al., which evaluated the frequency of JR, perceived benefits, and its developmental significance [16].

### 2.5. Study Process

Eligible participants received an electronic survey link and were able to complete the questionnaire at their convenience, either at home or in designated hospital areas. Informed consent and a brief overview of the study’s objectives were provided at the beginning of the survey to ensure voluntary participation and a clear understanding of the study’s purpose. Reliability and validity of the instruments were verified using Cronbach’s alpha. The CLS scale demonstrated a Cronbach’s alpha of 0.87, the JR scale demonstrated a Cronbach’s alpha of 0.79, and the CDM scale demonstrated a Cronbach’s alpha of 0.71, indicating acceptable internal consistency reliability. The threshold for statistical significance was maintained at the 0.05 level throughout all analyses.

### 2.6. Ethical Considerations

The study received approval from the institutional ethics committee (Permit No. 49/2024–2025). Participation was voluntary, and individuals were clearly informed that their choice to participate or decline would not affect their academic standing or employment. Personally identifiable information was never collected; anonymity was ensured throughout. All collected data were securely stored and exclusively utilized for research. The research team consistently upheld essential ethical standards, including informed consent, confidentiality, minimizing harm, and maintaining professional respect at every stage.

### 2.7. Data Analysis

The data analysis was carried out using IBM SPSS Statistics (Version 26) and IBM SPSS AMOS (Version 26). The initial phase involved a comprehensive cleaning of the dataset to ensure data accuracy and quality. Assumptions of normality were assessed before conducting ANOVA and structural equation modeling (SEM) analyses using skewness, kurtosis, and histogram distribution assessments. The findings indicated that the data were normally distributed and suitable for parametric analyses.

Descriptive statistics, including frequencies, percentages, means, and standard deviations, were computed to summarize participants’ demographic and occupational characteristics as well as the main study variables. Reliability analysis was performed for each study instrument individually using Cronbach’s alpha coefficients.

Inferential statistical analyses were conducted systematically to examine relationships among the study variables. ANOVA tests were used to assess associations between demographic variables and the main study variables, including JR, CLS, and CDM. Statistical significance was established at *p* < 0.05 with 95% confidence intervals (CIs). Multiple regression analyses were subsequently performed to examine the direct relationships between CLS and CDM.

To evaluate the mediating role of JR, SEM was conducted using IBM SPSS AMOS Version 26. Maximum likelihood estimation was employed to estimate model parameters, and bootstrapping procedures with 5000 resamples were performed to assess indirect mediation effects and generate bias‐corrected CIs. Model fit was evaluated using multiple goodness‐of‐fit indices, including the comparative fit index (CFI), Tucker–Lewis Index (TLI), root mean square error of approximation (RMSEA), and Chi‐square/degrees of freedom ratio (*χ* ^2^/df), based on recommended threshold values reported in the SEM literature. In addition, effect sizes were reported using eta squared (*η* ^2^) for ANOVA analyses and standardized beta coefficients (*β*) with 95% CIs for regression and SEM analyses.

## 3. Result

A total of 395 nurses responded. This number was higher than the minimum estimated sample size of 336. The response provided enough statistical power for the analysis. The majority of respondents belonged to the 31–40‐year age group (45.6%), followed by those aged 41–50 (32.7%). Males represented a slightly higher proportion (53.4%) compared to females (46.6%). In terms of marital status, 34.7% reported being married, 35.4% were single, and 29.9% were divorced. Educational attainment varied, with 52.9% possessing a bachelor’s degree, 29.1% holding a master’s degree, and 18.0% with a college diploma.

Regarding professional experience, 23.8% had between 11 and 15 years in practice, while 21.3% reported more than 20 years. Most respondents worked in emergency departments (33.4%), followed by ICUs (31.6%) and specialized units (18.2%). JR frequency showed considerable variation: 20.5% had not rotated, 27.3% had rotated once, 29.4% had rotated twice, and 22.8% reported three or more rotations. Among those with rotation experience, 44.6% had rotated between hospitals, 26.3% between departments, and 29.1% reported no exposure to rotation, as presented in Table 1.

**TABLE 1:** Distribution of demographic and occupational characteristics among the nursing staff (*N* = 395).

Variables	Category	*n*	%
Age category	22–30	78	19.70
	31–40	180	45.60
	41–50	129	32.70
	51–60	8	2.00

Gender	Female	184	46.60
	Male	211	53.40

Marital status	Married	137	34.70
	Single	140	35.40
	Divorced	118	29.90

Educational level	Bachelor’s	209	52.90
	Master’s	115	29.10
	College	71	18.00

Experience category	1–5	53	13.40
	6–10	82	20.80
	11–15	94	23.80
	16–20	82	20.80
	> 20	84	21.30

Department	Emergency department	132	33.40
	Intensive care unit	125	31.60
	Other specialized units	72	18.20
	Other	66	16.7

Job rotation frequency	None	81	20.50
	Once	108	27.30
	Twice	116	29.40
	Three times or more	90	22.80

Job rotation type	None	115	29.10
	Between hospitals	176	44.60
	Between departments	104	26.30

### 3.1. Associations Between Demographics and Study Variables

Table 2 presents the ANOVA test results comparing CLS, CDM, and JR based on various demographic variables. Specifically, a statistically significant difference was observed in CDM based on marital status (*F* = 3.261, *p* = 0.039, *η* ^2^ = 0.016), indicating a small effect size. Married participants demonstrated higher CDM scores compared with single and divorced participants. Educational level was also significantly associated with CDM (*F* = 5.239, *p* = 0.006, *η* ^2^ = 0.026), reflecting a small‐to‐moderate effect size. Participants holding master’s degrees reported higher CDM scores than those with bachelor’s degrees or college diplomas. In addition, gender differences in CDM were statistically significant (*F* = 9.002, *p* = 0.003, *η* ^2^ = 0.022), with male nurses demonstrating slightly higher decision‐making scores than female nurses. On the other hand, age, experience category, department, JR frequency, and JR type did not show any significant association with CDM (*p* > 0.05). Furthermore, none of the demographic variables were significantly linked to either CLS or JR, as all related tests produced nonsignificant results (*p* > 0.05). These findings indicate that CDM is influenced by certain demographic factors, namely, marital status, educational attainment, and gender, while CLS and JR appear unaffected by demographic characteristics.

**TABLE 2:** ANOVA test comparing clinical leadership skills, clinical decision making, and job rotation across demographic factors (*N* = 395).

Variables	Category	Clinical decision‐making				Clinical leadership skills				Job rotation			
		Mean (SD)	*F*	*p*	*η* ^2^	Mean (SD)	*F*	*p*	*η* ^2^	Mean (SD)	*F*	*p*	*η* ^2^
Age	22–30	3.00 (0.42)	1.096	0.351	0.008	3.13 (0.57)	1.187	0.314	0.009	3.02 (0.62)	1.646	0.178	0.012
	31–40	3.07 (0.44)				3.18 (0.56)				3.07 (0.67)			
	41–50	3.06 (0.40)				3.17 (0.55)				3.04 (0.75)			
	51–60	3.24 (0.55)				3.35 (0.46)				2.75 (0.74)			

Marital status	Married	3.11 (0.42)	3.261	0.039^∗^	0.016	3.18 (0.56)	0.759	0.469	0.004	3.04 (0.69)	0.006	0.994	0.000
	Single	3.07 (0.42)				3.15 (0.55)				3.03 (0.70)			
	Divorced	2.97 (0.43)				3.11 (0.58)				3.01 (0.73)			

Educational level	Bachelor’s	3.03 (0.41)	5.239	0.006^∗∗^	0.026	3.16 (0.54)	1.207	0.300	0.006	3.03 (0.66)	0.920	0.400	0.005
	Master’s	3.09 (0.46)				3.19 (0.58)				3.06 (0.69)			
	College	3.06 (0.43)				3.20 (0.54)				3.04 (0.78)			

Experience category	1–5	2.94 (0.41)	0.484	0.747	0.004	3.19 (0.59)	1.081	0.366	0.008	2.99 (0.61)	0.967	0.426	0.007
	6–10	3.05 (0.42)				3.11 (0.54)				3.17 (0.62)			
	11–15	3.10 (0.44)				3.22 (0.54)				2.98 (0.69)			
	16–20	3.05 (0.44)				3.13 (0.57)				3.02 (0.67)			

Department	Emergency department	3.01 (0.42)	0.337	0.799	0.003	3.14 (0.57)	0.823	0.482	0.006	3.10 (0.62)	1.596	0.190	0.012
	Intensive care unit	3.09 (0.45)				3.20 (0.56)				3.01 (0.68)			
	Specialized units	3.07 (0.42)				3.15 (0.55)				3.01 (0.72)			
	Other	3.06 (0.40)				3.22 (0.54)				3.01 (0.69)			

Job rotation frequency	None	2.99 (0.42)	1.165	0.323	0.009	3.16 (0.57)	0.806	0.491	0.006	3.04 (0.63)	0.882	0.450	0.007
	Once	3.07 (0.41)				3.15 (0.56)				3.04 (0.68)			
	Twice	3.06 (0.47)				3.20 (0.55)				3.05 (0.65)			
	Three times or more	3.10 (0.39)				3.19 (0.55)				3.03 (0.79)			

Job rotation type	None	3.00 (0.41)	0.395	0.674	0.002	3.16 (0.58)	0.750	0.473	0.004	3.09 (0.63)	0.728	0.484	0.004
	Between hospitals	3.06 (0.46)				3.17 (0.56)				3.00 (0.68)			
	Between department	3.10 (0.38)				3.20 (0.53)				3.05 (0.76)			

Gender	Female	3.04 (0.43)	9.002	0.003^∗∗^	0.022	3.16 (0.55)	2.511	0.114	0.006	3.04 (0.69)	0.935	0.334	0.002
	Male	3.07 (0.42)				3.19 (0.56)				3.05 (0.72)			

*Note:* *η* ^2^ = eta‐squared effect size.

^∗^ *p* < 0.05, *p* < 0.01.

^∗∗^ *p* < 0.001.

### 3.2. Structural Model Assessment

SEM indicated that CLS had a strong and statistically significant direct effect on CDM (*β* = 0.433, 95% CI [0.331, 0.535], *t* = 8.226, *p* < 0.001). In contrast, the direct effect of CLS on JR (*β* = 0.008, 95% CI [−0.041, 0.057], *t* = 0.325, *p* = 0.745) was not significant. Similarly, JR did not significantly predict CDM (*β* = 0.000, 95% CI [−0.052, 0.051], *t* = −0.010, *p* = 0.992), as presented in Table 3.

**TABLE 3:** Summary of structural model assessment direct effect.

Hypotheses	Relation	Original sample (*β*)	Standardized *β*	95% CI	*T*‐value	*p* value	Result
H1	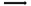 CLS CDM	0.433	0.383	[0.331, 0.535]	8.226	0.000^∗^	Accept
H2	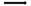 CLS JR	0.008	0.016	[−0.041, 0.057]	0.325	0.745	Reject
H3	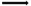 JR CDM	0.000	0.000	[−0.052, 0.051]	−0.010	0.992	Reject

*Note:* Bootstrapping procedures with 5000 resamples were applied.

^∗^ *p* < 0.001, *p* < 0.01, *p* < 0.05.

### 3.3. Mediation Analysis

Path analysis was conducted to examine whether JR mediated the relationship between CLS and CDM. CLS remained a statistically significant predictor of CDM (*β* = 0.433, 95% CI [0.331, 0.535], *p* < 0.001). However, the indirect effect through JR was not statistically significant (*β* = 0.035, 95% CI [−0.067, 0.128], *p* = 0.722). These findings indicate that JR did not act as a mediator between CLS and CDM in this cohort. Table 4 illustrates the structural path model, demonstrating a statistically significant direct pathway between CLS and CDM (*β* = 0.433, *p* < 0.001), while the pathways from CLS to JR (*β* = 0.008, *p* = 0.745), JR to CDM (*β* = 0.000, *p* = 0.992), and the indirect pathway (CLS ⟶ JR ⟶ CDM; *β* = 0.035, *p* = 0.722) were not statistically significant.

**TABLE 4:** Summary of path coefficients and direct and indirect effect testing (mediating results).

Hypothesis	Relation	Original sample (*β*)	Estimate	S.E.	C.R.	*T*‐value	95% CI	*p* value
Direct effect	CLS ‐> CDM	0.433	0.383	0.053	7.017	8.226	[0.331, 0.535]	0.000^∗^
Direct effect	CLS ‐> JR	0.008	0.016	0.024	3.562	0.325	[−0.041, 0.057]	0.745
Indirect effect	CLS ‐> JR ‐> CDM	0.035	0.384	0.099	7.025	0.356	[−0.052, 0.051]	0.722

*Note:* Bootstrapping procedures with 5000 resamples were applied.

^∗^ *p* < 0.001, *p* < 0.01, *p* < 0.05.

In summary, CLS emerged as a strong predictor of effective CDM, whereas JR neither directly predicted CDM nor mediated the relationship between CLS and CDM in this sample.

## 4. Discussion

This study confirmed that CLS were significantly and positively associated with nurses’ CDM within the Jordanian healthcare sector, and CLS demonstrated a significant direct association with CDM (*p* < 0.001). These findings are consistent with previous studies emphasizing the importance of leadership behaviors, including effective communication, problem‐solving, teamwork, and professional collaboration, in strengthening clinical judgment and CDM competencies among nurses [8, 17]. Effective nurse leaders are often characterized by their ability to make timely, accurate, and evidence‐based clinical decisions, particularly in complex healthcare environments such as emergency departments and ICUs [18]. These findings further support the perspective of Alilyyani et al., who suggested that nursing leadership competencies are developed through mentoring, professional interaction, experiential learning, and structured clinical practice rather than being solely innate characteristics [8]. Furthermore, regional nursing workforce literature has highlighted the importance of resilience, emotional engagement, and supportive organizational environments in strengthening nursing performance and professional competency development [19].

Moreover, the study findings demonstrated that JR did not play a statistically significant mediating role in the relationship between CLS and CDM, suggesting that CDM was more strongly associated with leadership competencies than with JR experiences within this sample. Therefore, the potential contribution of JR should be interpreted cautiously. This finding is partially consistent with the study by Gürsoy et al., which indicated that strengthening leadership orientations among nurses positively contributes to CDM skills through the integration of innovative educational and professional development strategies in nursing practice [20]. However, the current findings differ from some international organizational studies that reported positive effects of JR on professional competency development and workforce engagement. These inconsistencies may be explained by differences in healthcare systems, organizational culture, implementation frameworks, and mentoring availability across settings. Similarly, Alfuqaha et al. emphasized that the effectiveness of JR largely depends on structured implementation processes, organizational support, and clearly defined learning objectives [9]. Although rotation across diverse clinical units may enhance exposure, adaptability, and professional experience, the current findings suggest that staff movement alone may not be sufficient to improve CDM unless accompanied by structured mentorship, reflective learning opportunities, and competency‐based development frameworks [3, 21]. In addition, previous evidence suggests that poorly structured rotation systems may negatively affect continuity of care and team cohesion within healthcare settings [22]. Possible explanations for the nonsignificant mediation findings may include unstructured rotation programs, insufficient mentoring support, inconsistent implementation across departments, organizational barriers, workload pressures, and short rotation durations within participating hospitals.

The demographic data showed some interesting results. Certain demographic factors were able to predict CDM; they failed to predict leadership or JR outcomes. For example, male nurses had better scores in decision‐making, which goes along with the argument of gendered role expectations that may influence the level of confidence and the amount of authority one has in clinical contexts [17, 18]. In addition, married nurses could be reflecting a decision‐making ability of a higher level, as they have more life experience than the other group, due to varying family and personal responsibilities that may enhance their CDM [8]. Educational level was also shown to be related to decision‐making, where nurses with a master’s degree did better than those with only a bachelor’s degree, emphasizing the role of advanced education in developing the capacity for critical and evidence‐based practice [7, 23]. In contrast, the current findings indicated no statistically significant association between marital status and JR, which is consistent with the findings of Alfuqaha et al., who reported similar perceptions of JR among single and married nurses [24]. Furthermore, the absence of significant associations between demographic characteristics and CLS reinforces the perspective that leadership competencies are primarily developed through professional experience, mentorship, organizational culture, and continuous clinical engagement rather than demographic characteristics alone [8, 11]. These findings are also supported by regional nursing workforce literature emphasizing the importance of professional resilience, emotional engagement, and supportive work environments in strengthening nursing performance and professional competency development [19].

In summary, the present findings suggest that CLS were significantly associated with effective CDM, whereas demographic characteristics demonstrated comparatively limited associations. The findings further indicate that JR may contribute to nurses’ professional development only when implemented within structured leadership development frameworks that include mentorship, reflective practice, and competency‐based learning opportunities.

### 4.1. Limitation

Several limitations should be considered when interpreting the findings of this study. First, the cross‐sectional design limits the ability to establish causal relationships among CLS, JR, and CDM. Therefore, the identified associations should be interpreted cautiously, and causal inferences cannot be drawn. Future longitudinal or interventional studies are recommended to better examine the directionality and causal nature of these relationships. Second, data were collected using self‐reported questionnaires, which may have introduced social desirability bias and response bias, as participants may have provided responses perceived as professionally favorable rather than reflecting their actual experiences or behaviors. In addition, the use of online Google Forms for data collection may have contributed to selection bias by potentially limiting participation to nurses with greater internet accessibility, technological familiarity, or willingness to complete electronic surveys. Third, the study was conducted exclusively in governmental hospitals in Jordan, which may limit the generalizability of the findings to nurses working in private hospitals, military healthcare settings, or healthcare systems outside Jordan and similar Middle Eastern contexts. Furthermore, the study did not assess several organizational factors that may influence leadership development and CDM, such as organizational culture, mentoring quality, managerial support, staffing adequacy, and institutional leadership frameworks. Finally, because all study variables were collected from the same participants using the same data collection method at a single point in time, the possibility of common method bias cannot be excluded. Despite these limitations, the study provides important insights into the relationships among CLS, JR, and CDM among nurses within Jordanian governmental healthcare settings.

## 5. Conclusion

Leadership skills in the current study were significantly associated with nurses’ CDM, and leadership development may play a more substantial role in CDM than JR within this sample. Hence, for healthcare organizations in Jordan and similar areas, this evidence suggests that leadership development should be at the top of the agenda to reinforce nurses’ decision‐making skills and improve care quality. Thus, future research recommends longitudinal and interventional studies to further evaluate the role of structured JR programs in nursing leadership development.

### 5.1. Implication for Nursing Management

Healthcare leaders should invest in structured leadership development programs, including practical managerial recommendations. They structured mentorship, competency‐based rotation plans, leadership coaching, and reflective practice sessions. In addition, healthcare institutions should implement structured rotational frameworks aligned with safe patient care. All continuing and undergraduate education programs in nursing should include modules for developing leadership and decision‐making skills to encourage and promote students to develop their leadership skills in their careers.

## Author Contributions

Luma Ghazi Alzamel and Samar Thabet Jallad: conceptualization, methodology, data collection, formal analysis, writing–original draft, and writing–review and editing. Khitam Alsaqer and Maram Taher Alghabbashi: data collection, writing–original draft, and writing–reviewing and editing.

## Funding

This research received no specific grant from any funding agency in the public, commercial, or not‐for‐profit sectors. Funding for this research was covered by the author(s) of the article.

## Ethics Statement

The study received approval from the institutional ethics committee at Zarqa University (Permit No. 49/2024–2025). Information provided to the research team was anonymized prior to release from ZU. Informed consent was obtained for the use of these data.

## Consent

Informed consent and a brief overview of the study’s objectives were provided at the beginning of the survey to ensure voluntary participation and a clear understanding of the study’s purpose.

## Conflicts of Interest

The authors declare no conflicts of interest.

## Data Availability

The data that support the findings of this study are available from the corresponding author upon reasonable request, subject to institutional and ethical approvals. The data are not publicly available due to privacy and ethical restrictions.
